# Supplementary material for: Impact of Reaction Parameters and Water Matrices on the Removal of Organic Pollutants by TiO2/LED and ZnO/LED Heterogeneous Photocatalysis Using 365 and 398 nm Radiation
Source: Nanomaterials (Basel). 2021 Dec 21;12(1):5. doi: 10.3390/nano12010005 (PMC8746656; doi:10.3390/nano12010005)
Supplement: Supplementary file 1 [file nanomaterials-12-00005-s001.zip › nanomaterials-1474185-supplementary.pdf]

nanomaterials

Article

Impact of Reaction Parameters and Water Matrices on the Removal of Organic Pollutants by TiO_2/LED and ZnO/LED Heterogeneous Photocatalysis Using 365 and 398 nm Radiation

Máté Náfrádi [1], Tünde Alapi [1,*], Gábor Bencsik [2] and Csaba Janáky [2]

[1] Department of Inorganic and Analytical Chemistry, University of Szeged, Dóm tér 7., H-6720 Szeged, , Hungary; nafradim@chem.u-szeged.hu
[2] Department of Physical Chemistry and Materials Science, University of Szeged, Rerrich Béla tér 1., H-6720 Szeged, Hungary; bencsikg@chem.u-szeged.hu (G.B.); janaky@chem.u-szeged.hu (C.J.)
* Correspondence: alapi@chem.u-szeged.hu

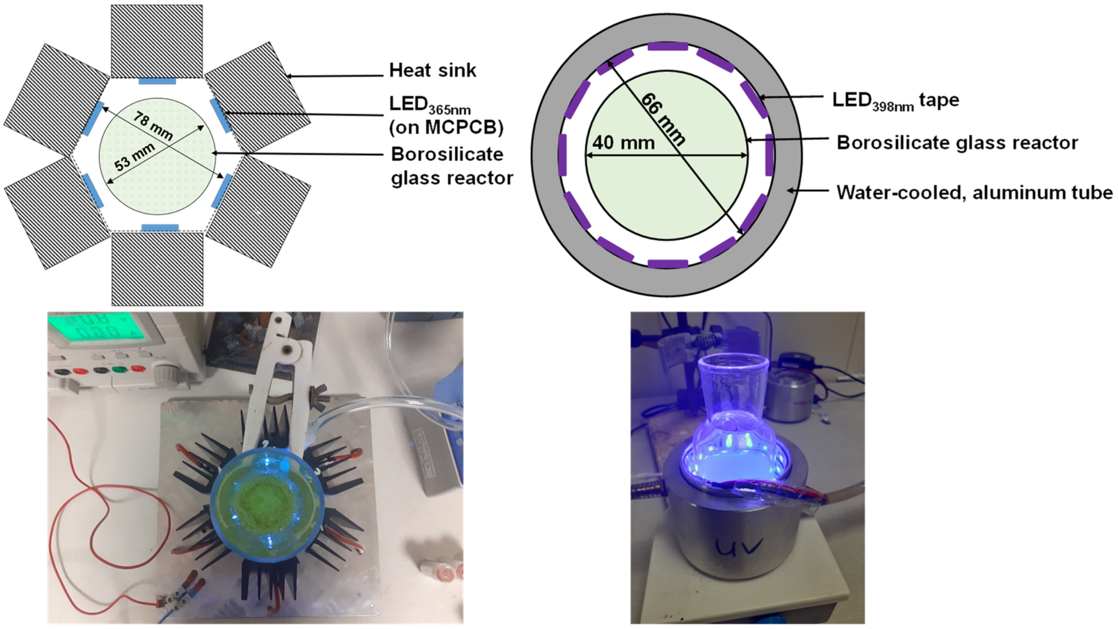

Figure S1. The schematic figures and photos of the photoreactors (**left**: LED_{365nm}, **right**: LED_{398nm}).

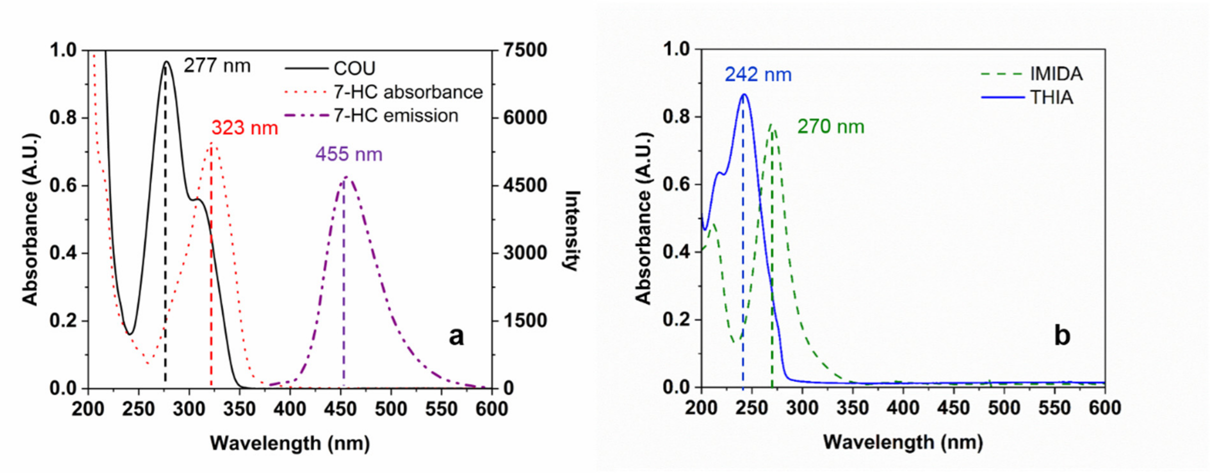

Figure S2. UV-Vis absorption and emission spectra of COU and 7-HC (**a**), and the UV-Vis absorption spectra of IMIDA and THIA (**b**).

Table S1. The list of used chemicals, their distributors and purity.

Chemical	Distributor	Purity
coumarin	Sigma Aldrich (St.Louis, USA)	99%
7-hydroxycoumarin	Sigma Aldrich (St.Louis, USA)	99%
imidacloprid	VWR (Radnor, USA)	98%
thiacloprid	Sigma Aldric h(St.Louis, USA)	99%
NaCl	VWR (Radnor, USA)	99%
$NaHCO_3^-$	VWR (Radnor, USA)	99%
HCl	Sigma Aldrich (St.Louis, USA)	98%
NaOH	VWR (Radnor, USA)	99%
Catalase (bovine liver)	Sigma Aldrich (St.Louis, USA)	>55% protein content
$Fe_2(SO_4)_3 \times nH_2O$	VWR (Radnor, USA)	98%
$K_2C_2O_4$	Reanal (Budapest, Hungary)	98%
1,4-phenantroline	Sigma Aldrich (St.Louis, USA)	99%
TiO_2	Acros Organics (Geel, Belgium)	99.5%
ZnO	Sigma Aldrich (St.Louis, USA)	80%
N_2	Messer Hungary (Budapest, Hungary)	99.995%
Synthetic air	Messer Hungary (Budapest, Hungary)	Medical grade
MeOH	VWR (Radnor, USA)	99.8%
H_2O	Merck-Millipore (Burlington, USA)	ultrapure

Table S2. The parameters of the matrices.

Parameter	Tap Water	Biologically Treated Domestic Wastewater
pH	7.4	7.8
Conductivity (µS cm^{-1})	627	1258
COD (mg dm^{-3})	4.2	24.4
NH_4^+–N (mg dm^{-3})	<0.4	<0.4
NO_3^- (mg dm^{-3})	<0.7	3.37
Cl^- (mg dm^{-3})	8.75	120
TOC (mg dm^{-3})	0.79	6.9
Inorganic carbon * (mg dm^{-3})	73.4	103.4

* Dissolved CO_2, HCO_3^- and CO_3^{2-}.

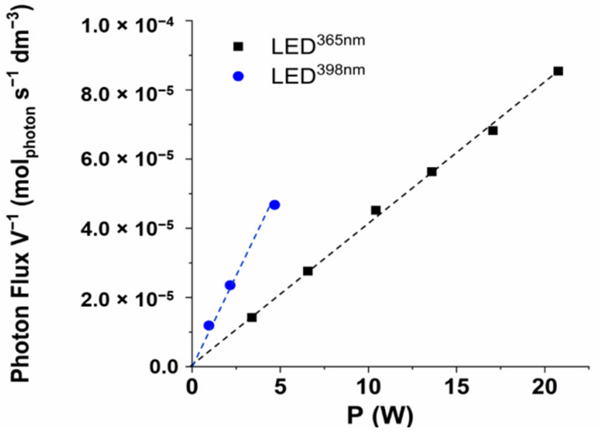

Figure S3. The photon flux of the LEDs as a function of electric power input.

Table S3. The photon flux of the light sources and the calculated electric efficiencies.

Light Source	Photon Flux (mol_{photon} s^{-1})	$P_{electric}$ (W)	E_{photon} * (eV)	$P_{radiant}$ (W)	Electric Efficiency $P_{radiant}/P_{electric}$
LED_{365nm}	2.83×10^{-6}	3.39	3.38	0.93	0.27
	5.52×10^{-6}	6.56	3.38	1.80	0.27
	1.13×10^{-5}	13.60	3.38	3.67	0.27
	1.71×10^{-5}	20.77	3.38	5.58	0.27
LED_{398nm}	1.19×10^{-6}	0.96	3.12	0.36	0.37
	2.35×10^{-6}	2.16	3.12	0.71	0.33
	4.68×10^{-6}	4.68	3.12	1.41	0.30

* Nominal value calculated for λ_{max}.

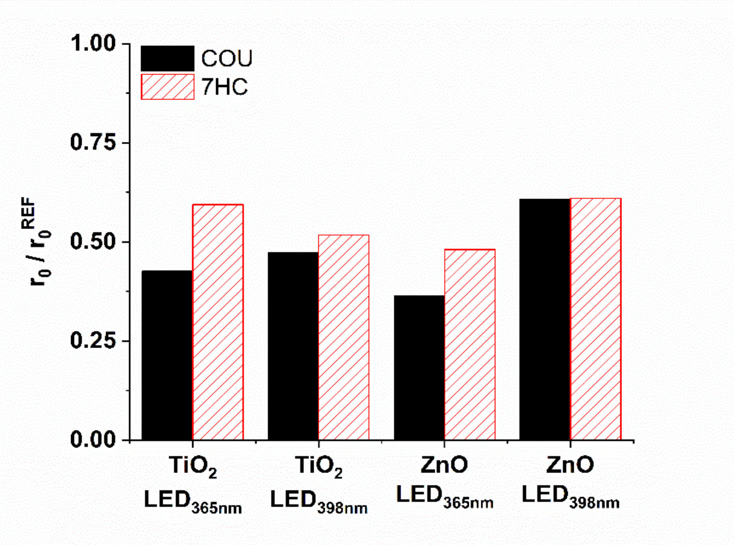

Figure S4. The effect of 5.0 × 10^{-3} M MeOH on the transformation rate of COU and the formation rate of 7-HC.

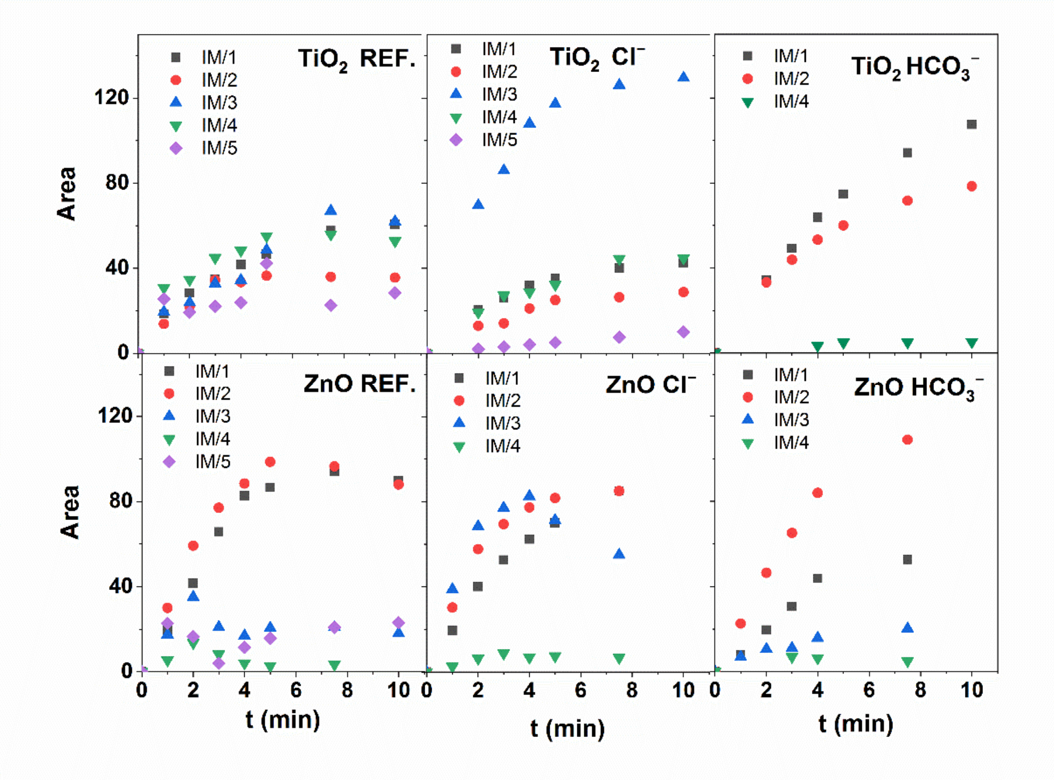

Figure S5. The effect of inorganic ions on the products of IMIDA detected by HPLC-DAD during treatment using LED_{365nm}.

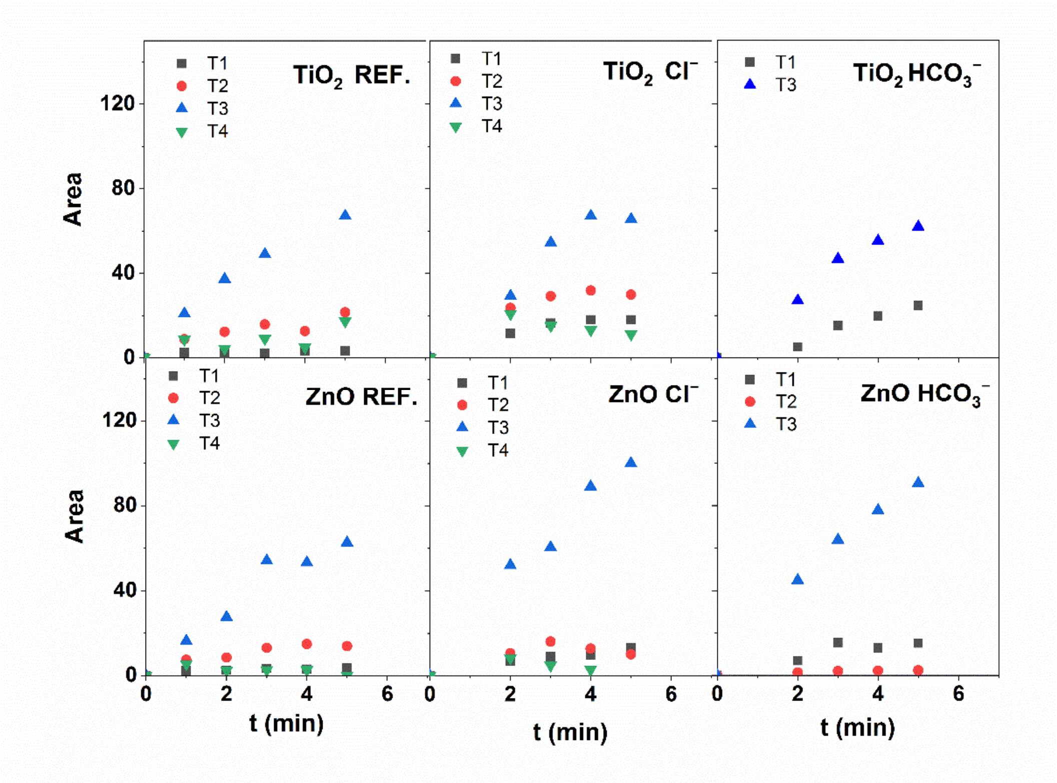

Figure S6. The effect of inorganic ions on the products of THIA detected by HPLC-DAD during treatment using LED_{365nm}.

Table S4. The inorganic standard electrode potentials of the ions and radical ions [105].

Half Reaction	Electrode Potential/V
$O_2(aq) + e^- \rightarrow O_2^{\bullet}$	−0.18
$O_2(aq) + H^+ + e^- \rightarrow HO_2^{\bullet}$	+0.10
$HO_2^{\bullet} + H^+ + e^- \rightarrow H_2O_2$	+1.46
$HO^{\bullet} + e^- + H^+ \rightarrow H_2O$	+2.730
$HO^{\bullet} + e^- \rightarrow OH^-$	+1.902
$Cl^{\bullet} + e^- \rightarrow Cl^-$	+2.432
$Cl_2^{\bullet -} + e^- \rightarrow 2\ Cl^-$	+2.126
$HOCl + e^- \rightarrow ClOH^{\bullet -}$	+0.25
$ClOH^{\bullet -} + e^- \rightarrow Cl^- + OH^-$	+1.912
$ClOH^{\bullet -} + e^- + H^+ \rightarrow Cl^- + H_2O$	+2.740
$NO_3^{\bullet} + e^- \rightarrow NO_3^-$	+2.466
$NO_2^{\bullet} + e^- \rightarrow NO_2^-$	+1.04
$CO_2^{\bullet -} + H^+ + e^- \rightarrow CO_3^{2-}$	+1.52
$CO_3^{\bullet -} + e^- \rightarrow HCO_2^-$	+1.57
